# Childhood Intussusception: A Literature Review

**DOI:** 10.1371/journal.pone.0068482

**Published:** 2013-07-22

**Authors:** James Jiang, Baoming Jiang, Umesh Parashar, Trang Nguyen, Julie Bines, Manish M. Patel

**Affiliations:** 1 National Center for Immunizations and Respiratory Diseases, Centers for Disease Control and Prevention, Atlanta, Georgia, United States of America; 2 The National Institute of Hygiene and Epidemiology, Hanoi, Vietnam; 3 Department of Paediatrics, University of Melbourne, Royal Children's Hospital, Murdoch Children's Research Institute, Melbourne, Australia; University of Ottawa, Canada

## Abstract

**Background:**

Postlicensure data has identified a causal link between rotavirus vaccines and intussusception in some settings. As rotavirus vaccines are introduced globally, monitoring intussusception will be crucial for ensuring safety of the vaccine programs.

**Methods:**

To obtain updated information on background rates and clinical management of intussusception, we reviewed studies of intussusception in children <18 years of age published since 2002. We assessed the incidence of intussusception by month of life among children <1 year of age, seasonality, method of diagnosis, treatment, and case-fatality.

**Findings:**

We identified 82 studies from North America, Asia, Europe, Oceania, Africa, Eastern Mediterranean, and Central & South America that reported a total of 44,454 intussusception events. The mean incidence of intussusception was 74 per 100,000 (range: 9–328) among children <1 year of age, with peak incidence among infants 5–7 months of age. No seasonal patterns were observed. A radiographic modality was used to diagnose intussusception in over 95% of the cases in all regions except Africa where clinical findings or surgery were used in 65% of the cases. Surgical rates were substantially higher in Africa (77%) and Central and South America (86%) compared to other regions (13–29%). Case-fatality also was higher in Africa (9%) compared to other regions (<1%). The primary limitation of this review relates to the heterogeneity in intussusception surveillance across different regions.

**Conclusion:**

This review of the intussusception literature from the past decade provides pertinent information that should facilitate implementation of intussusception surveillance for monitoring the postlicensure safety of rotavirus vaccines.

## Background

Intussusception is the invagination of one segment of the intestine within a more distal segment [1], [2]. It is the most common cause of bowel obstruction in infants, occurring usually between 4 and 10 months of age [3]. In most infants, the intussusception involves the ileum invaginating through the ileocecal valve into the cecum. As the bowel intussuscepts, it pulls along its blood supply. If the intussusception is not relieved, the vascular supply of the bowel may be compromised, resulting in intestinal ischemia and possibly perforation. Untreated intussusception may be fatal.

In 1999, an association was found between a tetravalent rhesus reassortant rotavirus vaccine (RotaShield) and intussusception in US infants prompting the manufacturer to withdraw the vaccine from the market [4], [5]. Because of this association, the two rotavirus vaccines that are currently recommended by the World Health Organization [RotaTeq (RV5), Merck & Co.; Rotarix (RV1), GSK Biologicals] were evaluated in large safety trials which excluded a risk of intussusception similar to that found with RotaShield (1 excess case per 10,000 vaccinated infants) [5], [6]. However, postlicensure studies in some high and middle income settings have identified a low level risk of 1–2 excess cases of intussusception per 100,000 infants after both RV1 and RV5 [7], [8]. These vaccines have now been introduced in some 45 countries worldwide, primarily in middle and high income countries, but broader introductions in low income countries of Africa and Asia are expected in next 3–5 years [9].

The past experience with RotaShield and the identification of a lower level risk of intussusception after RV1 and RV5 highlights the importance of ongoing intussusception monitoring as these new vaccines are broadly adopted in other regions of the world. In 2002, the WHO published a comprehensive literature review of childhood intussusception globally between the years 1960–2002 [3]. In anticipation of the global introduction of rotavirus vaccines, the objective of this literature review was to describe the epidemiology, diagnosis, and clinical manifestations of intussusception between the years 2002–2012. This review of the epidemiology of intussusception based on contemporary studies published in the last decade should serve as a valuable resource for implementing surveillance for intussusception to monitor the safety of these vaccines as they are broadly rolled out across the world.

## Methods

### Search strategy and selection criteria

We followed the PRISMA guidelines to identify all intussusception studies published between January 2002 (when the last review was conducted) and June 2012. We included all peer-reviewed articles and selected meeting abstracts. We searched PubMed MEDLINE and Google Scholar using the following keywords: “intussusception” or “intestinal invagination.” We included all human studies of intussusception among children <18 years of age published in English. Because we were interested in understanding the epidemiology of all intussusception events among children, full-text articles were not reviewed if they were case reports, animal studies, follow-up evaluation of chronic or recurrent intussusception, reports of intussusception events secondary to other conditions (e.g., vascular or congenital malformations, tumors), or reports of surgical or radiological management of intussusception. We also excluded those studies which did not include children <1 year of age. We reviewed the reference lists of all included articles to identify additional sources of data, missing articles, or meeting abstracts. Where there were multiple sources of data from the same study population, we relied on the data from the most complete peer-reviewed publication.

### Data Extraction and Management

Data from included studies were extracted onto a standardized table by JJ and MP. Reviewers were not blinded to study authors, affiliations, or journal name. Variables recorded from each article included, study locations (city & country), sample size, study period, age at diagnosis, month of presentation, diagnostic modality, treatment, death, and incidence.

### Data Analysis

We grouped data according to seven geographic regions of the world: Africa, Asia, Central & South America, Eastern Mediterranean, Europe, North America, and Oceania. Because of heterogeneity in under 5 mortality rates (u5MR) in Asia, we grouped Asian countries into high u5MR and low/very low u5MR according to WHO classification [10]. We present data on incidence of intussusception among children<1 year of age. We averaged the annual rate when rates for multiple years were presented individually. Among countries reporting incidence of intussusception, we were also interested in determining incidence of intussusception by month during the first year of life. To determine a pooled estimate of intussusception incidence by month of age globally, we extrapolated the proportion of intussusception events by month of age from studies that either presented monthly incidence or number of cases to the remaining studies that only presented intussusception incidence among children <1 year of age. While only 5 studies reported intussusception incidence by month of age, an additional 17 studies presented data on number of cases by month of age.

For studies reporting data on seasonality, we pooled data by geographic region on number of intussusception cases by calendar month. We also present pooled data on diagnostic modality, treatment, and prevalence of death by geographic region. For diagnosis and treatment, we excluded cases where this information was missing or unspecified.

All analyses were done with Microsoft EXCEL (Microsoft Corp, 2007).

## Results

**Figure 1:**
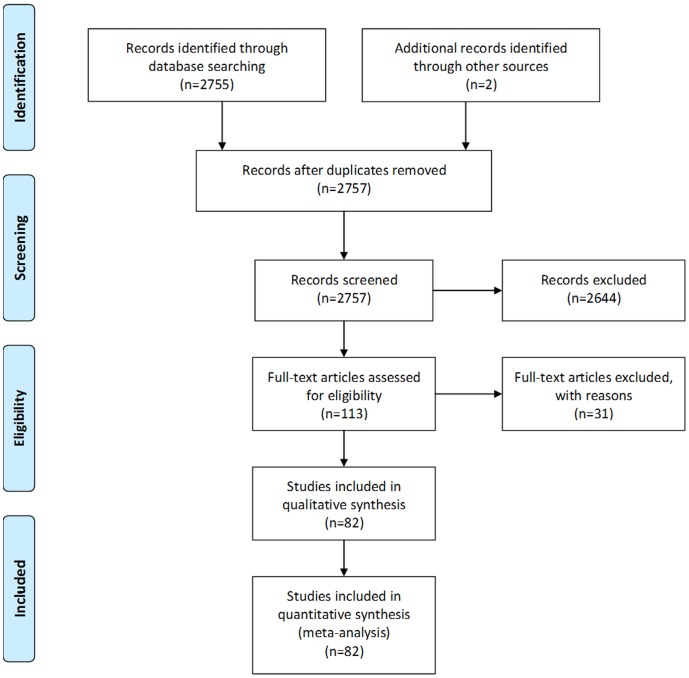
PRISMA flow diagram for the intussusception literature review.

We reviewed 2757 abstracts to identify a total of 113 potential full-text articles and 1 meeting abstract, from which 82 studies met the inclusion criteria for our analysis (Figure 1; Table 1) [11]–[91]. We excluded 31 articles due to non-English language (n = 8), duplicate data (n = 14), non-epidemiology study or review (6), included in previous review (2), or age <1 excluded (1). Included studies reported a total of 44,454 intussusception events from North America (n = 16,425), Asia: low or very low mortality (n = 14,382), Europe (n = 6,280), Oceania (2,761), Africa (2,895), Eastern Mediterranean (n = 644), and Central & South America (n = 538), Asia: high mortality (n = 356). Of these 82 studies, 44 (60%) were hospital-based studies, whereas 38 (40%) represented national or regional data on intussusception hospitalizations.

**Table 1:** List of all included studies.

Country	Study dates	National or Hospital based	No. of cases	Age, yrs	Ref.
**Africa**			**2895**		
Multiple^a^	1993–2003	International	1069	<1	{Steele, 2012 #92}
Kenya	2000–2003	Hospital	36	<6	{Kuremu, 2004 #55}
Kenya	1992–1999	Hospital	29	NR	{Muyembe, 2000 #65}
Ghana	2004–2007	Hospital	44	<13	{Abantanga, 2008 #1}
Ghana	2008–2009	Hospital	77	<5	{Enweronu-Laryea, 2012 #35}
Nigeria	1980–1998	Hospital	80	<12	{Ameh, 2002 #3}
Nigeria	1988–1998	Hospital	33	<1	{Ameh, 2002 #4}
Nigeria	1989–1998	Hospital	89	<1	{Archibong, 2001 #5}
Nigeria	1995–2001	Hospital	174	<7	{Bode, 2008 #12}
Nigeria	1996–2005	Hospital	38	<15	{Ogundoyin, 2009 #69}
Nigeria	1998–2003	Hospital	29	<8	{Edino, 2003 #30}
Nigeria	1998–2007	Hospital	87	<8	{Ekenze, 2010 #31}
Nigeria	2008–2009	Hospital	20	<2	{Ekenze, 2011 #32}
So. Africa	1998–2003	9 Hospitals	423	<14	{Moore, 2010 #63}
So. Africa	1996–2001	Hospital	106	NR	{Wiersma, 2004 #112}
Tanzania	2000–2004	Hospital	28	<10	{Carneiro, 2004 #21}
Tunisia	1984–2003	National	533	<5	{Chouikha, 2009 #27}
**Asia–high u5MR**			**356**		
Bangladesh	2001–2006	Regional	4	<2	{Zaman, 2009 #109}
India	2000–2003	Hospital	5	<1	{Bahl, 2009 #7}
India	2001–2004	Hospital	31	<5	{Bhowmick, 2009 #8}
India	1991–2000	Hospital	137	<5	{Raman, 2003 #75}
India	––	Hospital	179	NR	{Ramachandran, 2008 #74}
**Asia–low & very low u5MR**			**14382**		
China	2004–2009	Hospital	56	<14	{Zhang, 2011 #110}
Hong Kong^b^	1997–2003	National	531	<5	{Hong Kong Intussusception Study, 2007 #44}
Hong Kong^b^	1997–1999	National	190	<5	{Nelson, 2002 #67}
Japan	2007–2008	National	2427	<18	{Takeuchi, 2012 #93}
Japan	1978 – 2002	Hospital	91	<5	{Nakagomi, 2006 #66}
So. Korea	2000–2002	Regional	408	<5	{Jo, 2009 #47}
Malaysia	2000–2003	Regional	62	<5	{Giak, 2008 #40}
Singapore	1997–2007	National	217	<2	{Tan, 2009 #94}
Taiwan	1998–2007	National	8217	<15	{Chen, 2010 #24}
Thailand	1992–2009	Hospital	737	NR	{Kruatrachue, 2011 #54}
Thailand	2001–2006	5 hospitals	112	<5	{Khumjui, 2009 #51}
Thailand	2000–2005	Hospital	94	<14	{Pruksananonda, 2007 #73}
Uzbekistan	2004–2008	Regional	67	<2	{Latipov, 2011 #58}
Vietnam	2002–2003	Hospital	640	<2	{Justice, 2007 #50}
Vietnam	2002–2004	Hospital	533	<2	{Bines, 2006 #9}
**Central & South Americas**			**538**		
Latin America	n/a	International	222	<2	{Abate, #111}
Chile	2000–2001	Regional	95	<2	{O'Ryan, 2003 #71}
Panama	1998–2002	National	103	<1	{Saez-Llorens, 2004 #80}
Trinidad	2000–2007	Hospital	65	<3	{Tota-Maharaj, 2010 #98}
Venezuela	1998–2001	Regional	53	<1	{Perez-Schael, 2003 #72}
**Eastern Mediterranean**			**644**		
Saudi Arabia	1993 – 2000	Hospital	34	<8	{Al-Malki, 2005 #2}
Saudi Arabia	1984–2000	Hospital	37	<3	{Crankson, 2003 #29}
Israel	1990–2004	Hospital	316	<5	{Greenberg, 2008 #41}
Israel	2990–2002	Hospital	148	<5	{Eshed, 2003 #36}
Jordan	1979–2003	Hospital	109	<14	{Saleem, 2008 #81}
**Europe**			**6280**		
Austria	1999–2006	Hospital	111	<10	{Saxena, 2007 #85}
Denmark	1980–2001	National	1814	<5	{Fischer, 2004 #39}
Finland	2001–2006	National	53	<13	{Lappalainen, 2012 #57}
Germany	2006–2007	Regional	169	<1	{Wei, 2011 #102}
Germany	2006–2007	National	1200	<15	{Jenke, 2011 #46}
Germany	2005–2006	Regional	518	<17	{Kohl, 2010 #53}
Ireland	1998–2010	Hospital	256	<12	{Tareen, 2011 #96}
Ireland	1990–2000	Hospital	24	<2.5	{Hilal, 2002 #42}
Russia	1994–2005	Hospital	280	<14	{Shapkina, 2006 #86}
Spain	unspecified	Hospital	151	<12	{Rubi, 2002 #78}
Switzerland	2003–2006	National	294	<18	{Buettcher, 2007 #17}
Turkey	1991–2007	Hospital	105	<15	{Sonmez, 2012 #91}
Turkey	1993–2003	Hospital	179	NR	{Yalcin, 2009 #107}
UK/Ireland	2008–2009	National	261	<1	{Samad, 2012 #83}
UK/Ireland	1997 – 1998	Hospital	32	<1.5	{Willetts, 2001 #104}
England	1993–1995	National	833	<1	{Gay, 1999 #113}
**North America**			**16425**		
Canada	1993–2001	Regional	961	<6	{Somme, 2006 #90}
US	2000–2009	National	10,836	<1	{Yen, 2012 #108}
US	2001–2005	National^c^	22	<1	{Eng, 2012 #34}
US	2002–2012	Hospital	405	<7	{Fike, 2012 #38}
US	1996–2007	Regional	188	<17	{Shekherdimian, 2011 #87}
US	2002–2005	Military hospitals	293	<5	{Nylund, 2010 #68}
US	2001–2006	3 Hospitals	183	<1	{Cortese, 2009 #28}
US	1993–2004	National	3463	<1	{Tate, 2008 #97}
US	2001–2004	Hospital	26	<14	{Burjonrappa, 2007 #18}
Mexico	1999–2001	Regional	48	<1	{Velazquez, 2004 #100}
**Oceania**			**2761**		
Australia	2001–2009	Regional	197	<2	{Lloyd-Johnsen, 2012 #59}
Australia	2002–2004	Hospital	51	<2	{Bines, 2006 #9}
Australia	1994–2004	Regional	147	<12	{Blanch, 2007 #11}
Australia	1993–2003	Regional	23	<18	{Webby, 2006 #101}
Australia	1994–2000	National	1794	<1	{Justice, 2006 #49}
New Zealand	1998–2007	Hospital	189	<14	{Kodikara, 2010 #52}
New Zealand	1998–2003	National	277	<3	{Chen, 2005 #25}
New Zealand	1987–1998	Regional	83	NR	{Reid, 2001 #76}
**Intercontinental/Global**			**173**		
India, Brazil, Egypt Kenya	2004–2006	International	173	<10	{Awasthi, 2009 #6}

a: Botswana, Cote D'Ivoire, Ghana, Kenya, Nigeria, South Africa, Tanzania, Zambia, Zimbabwe.

b: these 2 studies had overlapping study years in same location but were included because data on age and clinical treatment were split.

c: authors randomly sampled a cohort of 100,000 infants from a national insurance claims database.

**Figure 2:**
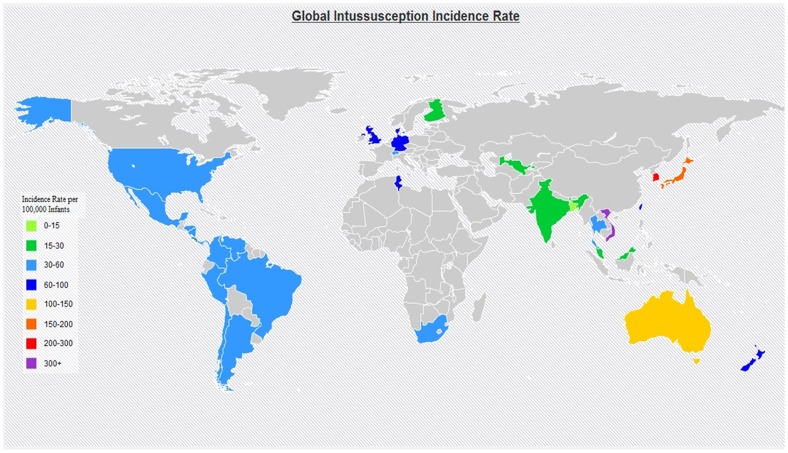
Global map of intussusception incidence. In countries where local, regional, and national studies were conducted, we preferentially mapped the national rates. For countries with more than one national study, average rates of the studies were used for the map. If no national rates were available, regional and/or local rates were used for the map.

Five reports presented incidence data by month of age, 17 studies presented data on number of cases by month of age and the incidence among children <1 year of age, and 13 only reported aggregate intussusception incidence among children <1 year of age. Among studies (n = 35) reporting incidence of intussusception among children <1 year of age, the mean incidence was 74 per 100,000 infant years (range: 9–328) (Table 2). While incidence in a majority (83%) of the studies was <100 per 100,000, higher incidence was observed in some populations including: Australia (101), Hong Kong (108), Japan (185), Israel (219), Vietnam (302), and South Korea (328) (Figure 2). Incidence of intussusception was <20 per 100,000 in some populations from Finland (20), India (18), Malaysia (18), and Bangladesh (9).

**Table 2:** Intussusception incidence among children <1 year of age by region.

Continent	Country	National/Regional/Hospital	Incidence per 100,000 <1 Yr.	Rates stratified by month of age
**Africa**	South Africa	National	56^a^	No
**Asia (high u5MR)**	Bangladesh	Regional	9	No
	India	Regional – Delhi	18	No
	Tunisia	National	62	No
**Asia-low/very low u5MR**	Hong Kong	National	108	No
	Japan	National	185^b^	No
	South Korea	Regional (Jeonbuk Province)	328^c^	No
	Malaysia	National	18	No
	Singapore	National	51	No
	Taiwan	National	77	No
	Thailand	National	36	No
	Uzbekistan	Regional – Bukhara	23	No
	Vietnam	Regional	302	No
**Central & South America**	11 countries	11 countries	51	No
	Chile	Regional – Santiago	51	No
	Panama	National	30	No
	Venezuela	Regional – Carabobo	35	No
**Eastern Mediterranean**	Israel (Jewish)	Hospital	219	No
	Israel (Bedouin)	Hospital	75	No
**Europe**	Denmark^d^	National	66	Yes
	Finland	National	20	No
	Germany^d^	Regional – Bavaria & NRW	62	Yes
	Germany	National	60	No
	Germany	Regional – Bavaria	72	No
	Switzerland	National	38	No
	England^d^	National	66	Yes
**North America**	US^d^	National	33	Yes
	US	National	33	No
	US	3 hospitals	49	No
	US^d^	National	34	Yes
**Oceania**	Australia	Regional – Melbourne	27	No
		Hospital	71	No
		Regional – Northern Territory	65	No
		National	101	No
	New Zealand	National	65	No
**ALL REGIONS (Average)**			**74**	**No**

a: rate for <2 year olds in the paper; we recalculated rate for <1 year of age based on presented data.

b: midpoint of the range presented in the study.

c: rates presented for <6 and 6–11 months in the paper; we recalculated rates for <1 based on these data.

d: rates by age in months for the most recent surveillance years 1995–1999 were obtained from figure using Digitize-It; mean incidence for <1 presented {Fischer, 2004 #39}.

**Figure 3:**
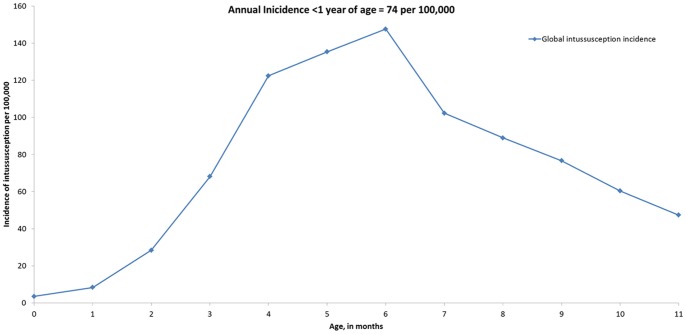
Incidence of intussusception by month of life during first year of life. Intussusception incidence or number of cases by month of life from 22 studies extrapolated to remaining 13 studies that only presented data on rates for infants <1 year (as displayed in Table 2).

Five studies reported incidence of intussusception by month of age and 15 reported number of cases by month of age among children <1 year of age. We extrapolated the proportion of intussusception incidence by month of age relative to the mean incidence among children <1 year from the 5 studies to the 30 studies that only reported incidence among children <1 year (Figure 3). The age distribution of intussusception incidence and number of cases were similar, with lowest incidence among children 0–2 months of age (13–37 per 100,000) and peak incidence among those 4–7 months of age (97–126 per 100,000).

**Figure 4:**
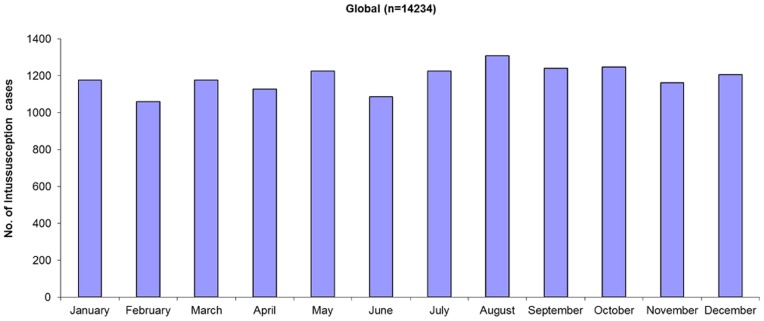
Seasonality of intussusception globally.

**Figure 5:**
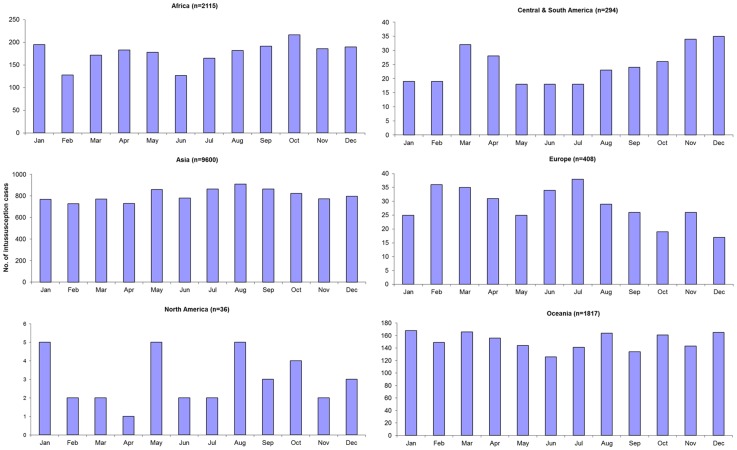
Seasonality of intussusception, by WHO region.

Distribution of intussusception cases by calendar month of year was reported in 21 studies (Figure 4). No particular seasonal pattern in intussusception was seen globally or in any of the regions except North America (Figure 5); however, only 1 small study of 36 cases provided data on seasonality of intussusception in North America [82].

**Figure 6:**
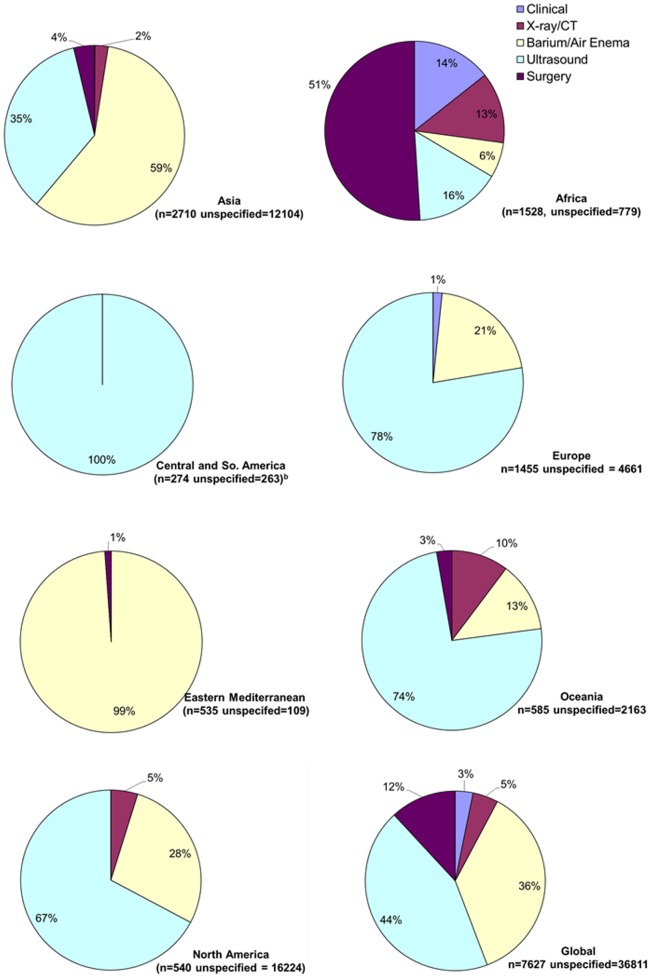
Diagnosis of intussusception by WHO region. Studies where diagnosis was unspecified were not included. Data from Central and South America were from one prospective research study that might not reflect clinical practice under routine conditions.

**Figure 7:**
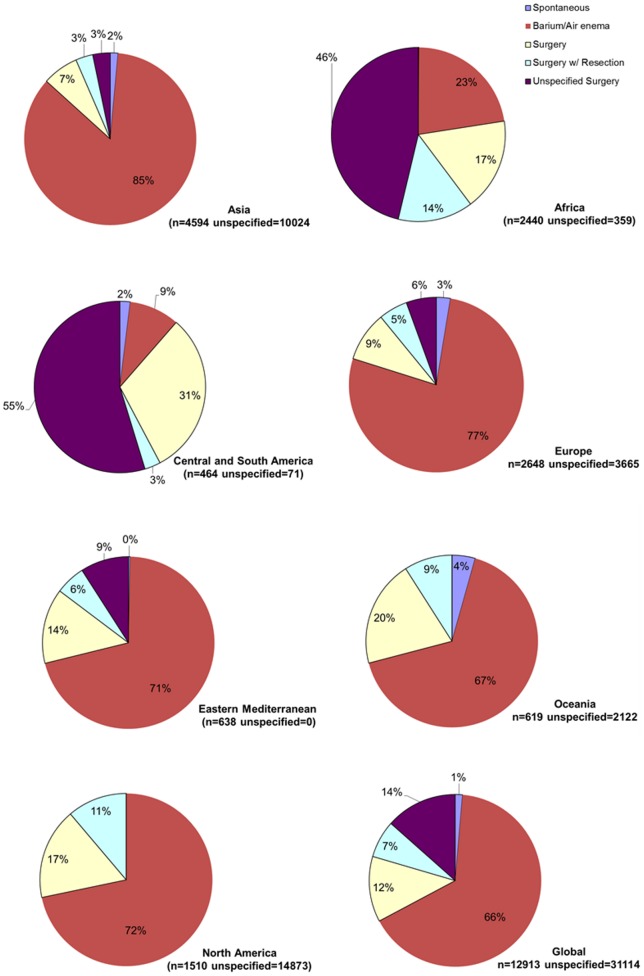
Treatment of intussusception by WHO region. Studies where treatment was unspecified were not included.

Diagnosis of intussusception varied substantially by region. Diagnosis of intussusception was made by a radiographic modality (e.g., air-contrast enema, ultrasound, or computed tomography) in 95%–100% of the cases from all regions of the world except Africa where intussusception was diagnosed by clinical findings or surgery in 65% of the case; however, data on method of diagnosing intussusception were limited in countries from Eastern Mediterranean, Americas, and Oceania (Figure 6). Treatment of intussusception also differed by region (Figure 7), with higher prevalence of surgery in Central/South America (86%) and Africa (77%) compared to Eastern Mediterranean (29%), Oceania (29%), North America (28%), Europe (20%), and Asia (13%).

The proportion of children with intussusception who died after presenting to the hospital was substantially higher in Africa (9.4%; range  = 2–25) compared to Asia (0.2%; range  = 0–6), Central and South America (0.6%; range  = 0–1), Eastern Mediterranean (0.8%; range  = 0–5), Europe (0.1%; range  = 0–1), North America (0.4%; range  = 0–1); and Oceania (0%; range 0–0.1%) (Table 3).

**Table 3:** Case-fatality among children hospitalized with intussusception.

Country	No. of IS cases	Deaths	
		No.	%
**Total (Africa)**	**1961**	**185**	**9.4%**
*Multi-country*: Botswana, Cote D'Ivoire, Ghana, Kenya, Malawi, Nigeria, South Africa, Tanzania, Zambia, Zimbabwe	863	108	12.5%
Kenya	36	5	13.9%
	29	5	17.2%
Ghana	44	1	2.3%
Nigeria	33	6	18.2%
	89	2	2.2%
	174	21	12.1%
	29	4	13.8%
	87	7	8.0%
	20	0	0.0%
South Africa	423	9	2.1%
	106	10	9.4%
Tanzania	28	7	25.0%
**Total (Asia)**	**4323**	**8**	**0.19%**
China	56	0	0.0%
India	31	0	0.0%
	137	1	0.7%
	179	0	0.0%
Japan	2427	2	0.1%
Korea	408	1	0.2%
Malaysia	62	0	0.0%
Singapore	217	0	0.0%
Thailand	112	0	0.0%
	94	0	0.0%
Uzbekistan	67	4	6.0%
Vietnam	533	0	0.0%
**Total (Central and South America)**	**538**	**3**	**0.6%**
Latin America	222	2	0.9%
Chile	95	0	0.0%
Panama	103	1	1.0%
Trinidad & Tobago	65	0	0.0%
Venezuela	53	0	0.0%
**Total (Eastern Mediterranean)**	**644**	**5**	**0.8%**
Saudi Arabia	34	0	0.0%
	37	0	0.0%
Israel	316	0	0.0%
	148	0	0.0%
Jordan	109	5	4.6%
**Total (Europe)**	**2588**	**3**	**0.1%**
Austria	111	0	0.0%
Germany	1189	0	0.0%
Ireland	256	0	0.0%
	24	0	0.0%
Russia	280	0	0.0%
Spain	151	1	0.7%
Turkey	105	1	1.0%
	179	0	0.0%
UK/Ireland	261	1	0.4%
	32	0	0.0%
**Total (No. America)**	**3860**	**16**	**0.4%**
US	188	0	0.0%
	183	2	1.1%
	3463	14	0.4%
	26	0	0.0%
**Total (Oceania)**	**2738**	**1**	**0.0%**
Australia	51	0	0.0%
	197	0	0.0%
	147	0	0.0%
	1794	1	0.1%
New Zealand	189	0	0.0%
	277	0	0.0%
	83	0	0.0%

## Discussion

A key issue for rotavirus immunization programs in the postlicensure era is the need for safety monitoring with regard to intussusception [2], [5], [92]. Our review of the intussusception literature from the past decade provides pertinent information that should facilitate implementation of intussusception surveillance for monitoring the postlicensure safety of rotavirus vaccines.

First, we demonstrate that natural intussusception is a very rare condition in most regions of the world, particularly among infants <3–4 months of age when the first dose of rotavirus vaccine is typically administered. Because any potential risk with rotavirus vaccines is greatest after the first dose of vaccine which is typically recommended to be administered at 6–15 weeks of age, [5] the lower rates of intussusception in this age group suggests that timely administration of vaccine would minimize the attributable risk of vaccine [93]. That is, the higher rates of intussusception among infants older than 3–4 months indicates that any vaccine associated risk of intussusception could lead to more cases of intussusception associated with vaccine in settings where delays in vaccination are common. If vaccine is linked to a potential risk of intussusception, age stratified data on intussusception are necessary for interpreting these estimates of vaccine-associated risk and calculating the number of cases that are potentially attributable to vaccine after the implementation of a vaccine program [93]. The age-stratified data in this review will be a valuable resource for conducting benefit risk analyses, should a rotavirus vaccine program be linked to any potential risk of intussusception.

Second, the incidence of intussusception varies substantially by region, thus highlighting the importance of the need for regional baseline data when evaluating postlicensure trends of intussusception for assessing any potential vaccine-associated risk. Risk of intussusception after the current rotavirus vaccines has also varied by region. In Mexico and Australia, postlicensure studies have identified an increased low-level risk of intussusception after the first dose of both rotavirus vaccines, ranging from 1 to 2 cases of intussusception per 100,000 vaccinated infants [7], [8]. While an early study in the United States did not identify a risk of intussusception after RV5 [94], a recent study has shown that a low level risk of intussusception is likely to exist with both vaccines [95]. However, a similar level of risk was not observed in Brazil after RV1 [7]. While these differences in risk might be related to a chance finding, effect modification of risk related to an environmental or genetic factor that differs between the populations cannot be excluded. In our review, we observed a wide range in the incidence of intussusception globally. The cause of natural intussusception in a majority of infants is not known and has been previously reviewed. Differences in infant diet, breastfeeding, maternal antibody levels, prevalence of enteropathogens (e.g., respiratory adenovirus, rotavirus) might all contribute to the differences in background rates of intussusception [3], [96]. It remains unclear whether populations with higher background risk of intussusception also would carry a higher risk of vaccine-associated risk. However, any potential risk of intussusception associated with vaccine in populations with higher background risk would lead to higher number of excess cases that would be attributable to vaccine.

Third, the clinical management of intussusception in Africa differs markedly from other regions of the world where data exist, predominantly relying on clinical manifestations for diagnosis and surgery for treatment. The paucity of data on method of diagnosing intussusception from Eastern Mediterranean, Americas, and Oceania region limits the generalizability of this information in countries from these regions. In addition, limited published data are available on clinical management and outcome of intussusception in high mortality Asian countries but it would seem that surgical rates are likely to be higher than the lower mortality Asian countries. The differences in clinical management of intussusception in high mortality settings compared to low mortality settings emphasizes the need for augmenting surveillance practices depending on the location. For example, strengthening collaborations with surgeons to improve case-capture should be prioritized when monitoring the postlicensure safety of rotavirus vaccines in Africa. As rotavirus vaccines are more broadly introduced into immunization programs in Africa and Asia, these current data on the epidemiology and clinical management of intussusception should facilitate the future implementation of intussusception surveillance and inform benefit risk calculations for defining policy and decision making.

Studies assess the role of wild-type rotavirus infection as a cause of intussusception have led to conflicting findings with several studies suggesting that a significant association is unlikely [3]. The absence of any marked seasonal patterns in intussusception, particularly in settings of Europe, Oceania, and Central and South America where rotavirus disease is quite seasonal supports the contention that rotavirus is unlikely to be a prominent etiologic cause of natural intussusception. This has some implications for postlicensure monitoring because attempts have previously been made to assess whether rotavirus vaccination would have a protective effect against intussusception vis-à-vis protecting against wild-type rotavirus infection. Establishing whether vaccine confers protection against intussusception might be challenging if only a small etiologic fraction of naturally occurring intussusception is related to wild-type rotavirus infection [5], [97].

Our review provides background rates of intussusception against which rates of intussusception after the introduction of vaccine could be compared for assessing potential vaccine-associated risk. However, the paucity of background rates from most settings supports the need for relying on other analytic methods of safety monitoring after the introduction of rotavirus vaccine [2]. The self-controlled case-series method has now been successfully applied in several settings for assessing a potential link between vaccine and intussusception [7]. This method relies on active, hospital based surveillance of intussusception and does not require information on background rates of intussusception in the population under surveillance [98]. Our review provides important considerations when establishing surveillance for intussusception. First, in resource poor settings of Africa, specific attention should be placed on hospitals with surgery suites which are likely to manage most intussusception events. While background rates are not specifically needed for the case-series method, in settings where no background rates are available, some efforts could be made to establish population-based studies which would be necessary for determining the attributable risk of vaccine, should any increased risk be identified. Lastly, the varying incidence of intussusception during the first six months of life when rotavirus vaccines are administered strongly indicates the need for addressing confounding effects of age when determining whether a causal link exists between rotavirus vaccine and intussusception cases identified through surveillance.

Intussusception case-fatality was <1% in all developed countries of the world. However, the marked difference in case-fatality between Asia (<1%) and Africa (9%) was intriguing. This finding might reflect differences in healthcare infrastructure or delays in care, or perhaps might be related to a publication bias with a paucity of surveillance data in the poorest populations of Asia. Previous analyses of benefits versus risk of rotavirus vaccine have conservatively assumed intussusception case-fatality rate of 25% in Asia, which in light of this review is high and could be reconsidered for future benefit risk deliberations [93]. Even if case-fatality rates were to be higher than those identified in this review, some adjustment would be prudent for obtaining valid estimates of any potential risk associated with rotavirus vaccine in Asia.

Our review must be considered with some limitations. Because intussusception is a very rare condition, a fair amount of heterogeneity in surveillance practice is likely to exist across regions. In particular, establishing incidence of intussusception is rather challenging in many settings without established national or regional electronic records and thus published rates of intussusception might be prone to over or underestimation. The availability of a standardized case definition by the Brighton Collaboration reduces case misclassification but sensitivity of the case-definition might be reduced in resource poor settings where diagnostic modalities are not available and relying on clinical judgment and surgery is required for diagnosis [1]. The case-fatality from these hospital based surveillance studies in resource poor settings also certainly underestimates the true case-fatality because deaths are likely to occur out of hospital. While some 30 countries had introduced a rotavirus between 2006 and 2011, all but one of the studies included in this review [83] captured data before the introduction of a rotavirus vaccine.

In summary, this review of current epidemiology and clinical management of intussusception should prove to be a valuable resource for establishing intussusception surveillance and interpreting these surveillance data from various regions of the world where rotavirus vaccines are likely to be introduced in the next several years.

### Ethics statement

Because these are publicly available non-identifiable data, an ethics statement was not required for this work.

## Supporting Information

Checklist S1 **PRISMA Checklist for the preferred reporting list for the intussusception literature review.**
(DOC)
